# Live-cell imaging study of mitochondrial morphology in mammalian cells irradiated

**DOI:** 10.1093/jrr/rrt167

**Published:** 2014-03

**Authors:** Yukiko Kanari, Miho Noguchi, Kiichi Kaminaga, Yuka Sakamoto, Akinari Yokoya

**Affiliations:** 1 Ibaraki University, Mito, Ibaraki, Japan; 2 JAEA, Advanced Science Research Center, Tokai, Ibaraki, Japan

**Keywords:** mitochondria, cytoplasmic irradiation, FUCCI cells, live-cell imaging

## Abstract

Recent reports suggest that extranuclear targets in cytoplasm may have a role in mediating radiation effects in mammalian cells exposed to ionizing radiation. We have focused mitochondria as a target of ionizing radiation, particularly heavy ions, because mitochondria are a kind of organelles existing widely in cytoplasm. They play a vitally important role of ATP formation through the operation of electron transport chain located in the membranes, and generate reactive oxygen species as a by-product in the process of ATP production. As mitochondria are fusing or dividing depended on cell cycle, their morphology is continuously changing [ 1]. Defects of these processes contribute to the pathogenesis of neurodegenerative disease. Effects of high LET irradiation, such as heavy ion bombardment, on mitochondrial morphology, however, remain to be fully elucidated. The object of this study is to reveal effects of high LET radiation on mitochondria.

We have performed preliminary test experiments using X-rays. We used NMuMG (Normal murine mammary gland)-FUCCI2 cell line [ 2]. Using the FUCCI2-expressing cells, we can distinguish G1 or S/G2/M phase cells as observed red or green fluorescence in their nucleus. We also labeled mitochondria by another fluorescence probe, Mitotracker Red. Kinetics of mitochondrial morphology was analyzed by the live-cell imaging technique using a fluorescence microscope. Mitochondrial images were captured at certain hours after irradiation, and classified them into three categories, namely tubes, intermediates and fragments (Fig. 1).

We found that X-ray irradiation of cells caused mitochondrial fragmentation, and cell population with fragmented mitochondria increased with increasing dose, and also with time after irradiation (Fig. 1). Although the cells became confluent around 48 h after irradiation as indicated by the cell-nucleus color, the mitochondrial morphology was still changing. Particularly, the population of the cells with fragments showed a maximum at 96 h after irradiation when they were exposed to 8 Gy X-rays. Based on the present study, we further investigate morphological changes of mitochondria by high LET particle irradiation in future.

**Fig. 1.**
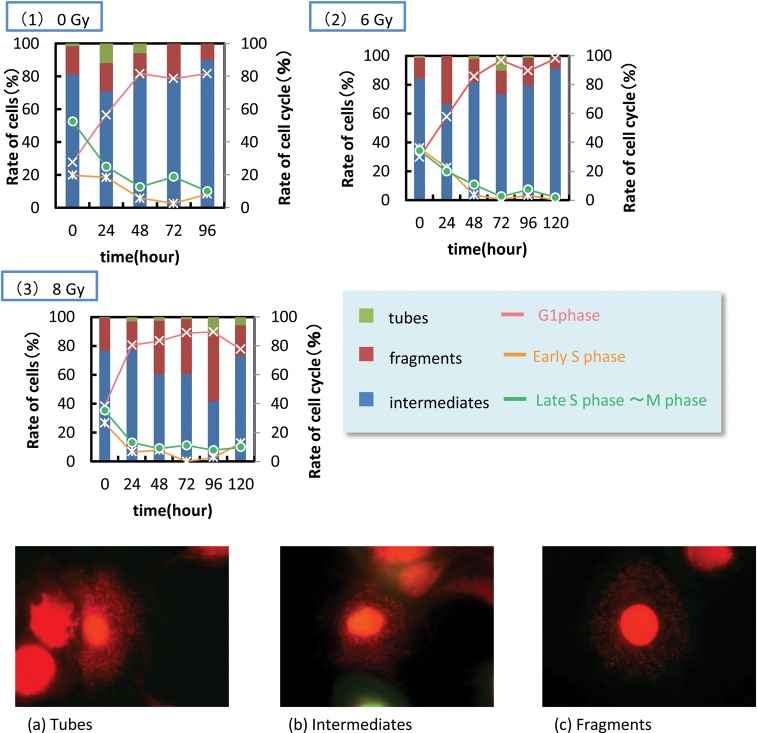
Relative fraction of cells showing each morphology for non-irradiated (control) and charectors of mitochondrial morphology. Irradiated with (1) 0Gy, (2) 6 Gy and (2) 8 Gy. The cell cycles are also shown by solid lines. (**a**) Mostly mitochondria look tubular. (**b**) Both tubular and fragment forms are observed with the same rate in a cell. (**c**) Mitochondria are visible as dots, and straggle in whole cytoplasm.

Relative fraction of cells showing each morphology for non-irradiated (control) and charectors of mitochondrial morphology. Irradiated with (1) 0Gy, (2) 6 Gy and (2) 8 Gy. The cell cycles are also shown by solid lines. (**a**) Mostly mitochondria look tubular. (**b**) Both tubular and fragment forms are observed with the same rate in a cell. (**c**) Mitochondria are visible as dots, and straggle in whole cytoplasm.
